# Model for Wireless Magnetoelastic Strain Sensors †

**DOI:** 10.3390/s20123557

**Published:** 2020-06-23

**Authors:** Eduardo S. Bastos, Cristina Bormio-Nunes, Thomas G. R. Clarke, Frank P. Missell

**Affiliations:** 1 Laboratório de Metalurgia Física, Universidade Federal do Rio Grande do Sul, Porto Alegre 91501-970, Brazil; eduardo.stimamiglio@ufrgs.br (E.S.B.); tclarke@demet.ufrgs.br (T.G.R.C.); 2 Escola de Engenharia de Lorena, Universidade de São Paulo, Lorena 12.602-810, Brazil; cristina.bormio@usp.br

**Keywords:** Fe–Al–B, magnetoelastic, magnetostriction, Metglas 2826MB3, steel, strain sensor

## Abstract

This paper describes a magnetoelastic strain sensor based on the ∆*E* effect and discusses some materials used in its construction. A polycrystalline Fe–Al–B alloy with good quality magnetoelastic properties was used as the transducer and glued to the test object, either brass plates or rods of SAE 1010 steel. The strain-dependent magnetic field of the transducer changes the operating point of the resonator, a strip of field-annealed Metglas 2826MB3, resulting in a modification of its resonant frequency. A model was developed to simulate the strain-dependent magnetic field acting on the resonator and thus to calculate curves of resonant frequency vs. deformation. With the help of this model, differences in the shape of the frequency vs. strain curve can be understood. For a sensor with resonant frequency of 60.5 kHz glued to a rod of SAE 1010 steel, a total resonant frequency variation ∆f ~7 kHz was observed for a deformation of 1100 ppm. The geometry of this sensor is especially favorable for the remote monitoring of a steel surface, such as the wires of the tensile armor of a marine riser.

## 1. Introduction

This paper is an extension of work originally presented at the 12th International Conference on Sensing Technology [1] in Limerick, Ireland. The goal here is to develop a model for the strain-dependent magnetic field of wireless magnetoelastic strain sensors on different surfaces, for example, on the tensile armor of a marine riser. Many other modern industrial applications of strain sensors also demand a contactless version of the sensor. For example, monitoring the structural health of large structures such as bridges frequently makes use of a wireless strain sensor. Magnetoelastic stress sensors on large steel cables of Qiangjiang No. 4 Bridge in China [2] are examples of noncontact stress monitoring for steel cables and prestressed tendons on suspension bridges, cable-stayed bridges, and other ferromagnetic structures. In a different sector, the measurement of torques on the steering column of an automobile for the purpose of servo control can be performed with strain gauges, which must, however, be controlled in a noncontact manner by a rotary transformer, which surrounds the steering column [3].

**Figure 1:**
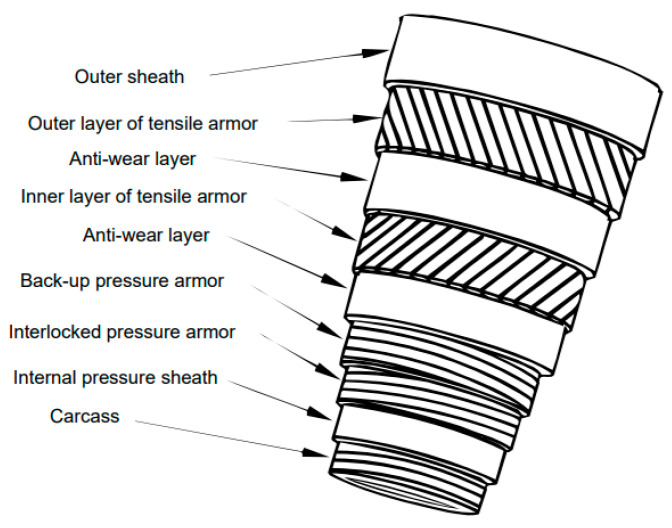
The several layers of a flexible marine riser. The outer sheath, antiwear layers, and internal pressure sheath are made from polymeric material, while the tensile armors, pressure armors, and carcass are constructed of steel [7].

In particular, the oil and gas industry demands noncontact solutions for solving difficult problems. For example, noncontact electromagnetic acoustic transducers (EMATs) are used to monitor corrosion in polymer-coated rigid risers [4], which are used for transporting petroleum products. In addition to the rigid risers, the flexible marine risers also call for imaginative solutions in the deployment of strain gauges. The most important difference between drilling a well in deep water and drilling on the surface is that, in the first case, there is a marine riser several thousand meters in length between the drilling platform and the well head. The riser is a multilayer, flexible tube, which is necessarily subject to maritime currents and is, therefore, constantly in motion [5,6]. The various layers of a flexible marine riser are shown schematically in Figure 1.

Of particular note are the two layers of tensile armor, each consisting of strips of steel wound in a spiral fashion. It would be desirable to constantly monitor these steel structural components, which are subject to fatigue wear and whose rupture would lead to extensive environmental damage. The construction of a riser with its polymer outer layer makes it difficult to monitor the steel strips that sustain the structure of the long tube. Traditional strain gauges [8] require extensive wiring and would be difficult to use in this application. Another technique that has been used to measure the deformation of the steel strips employs a Bragg grating engraved on the nucleus of an optical fiber [9]. For constant temperatures, the wavelength variation of the reflected light is proportional to the variation in the grating spacing [10]. A device for measuring small strains [10] employs a Bragg grating and rare-earth permanent magnets.

Magnetic techniques such as magnetic Barkhausen noise (MBN) or magnetic anisotropy and permeability system (MAPS) [11] could be used to determine residual stress and thereby furnish information about the integrity of the steel strips in the tensile armor of a riser. However, the amplitudes of the MBN signals are sensitive to the thicknesses of the outer sheath and insulating layers. The MAPS technique normally demands rotation of the sensor on the test specimen. Because that is not possible in this case, a line of sensors, each rotated slightly in relation to the previous sensor, might be used to shed light on the strain state of the steel strips that make up the tensile armor [11]. 

The present paper discusses materials and functioning of a magnetoelastic strain sensor [12,13] based on the ∆*E* effect [14], which is caused by the variation of the Young’s modulus of a ferromagnetic material due to a magnetic field. The resonant frequency of amorphous strips may be interrogated remotely via magnetic fields or other methods [15,16,17,18,19], making it possible to apply the strips as sensors in situations that do not permit direct access to the measuring surface. The ∆*E* effect is also the principle behind [20] electronic article surveillance (EAS) technology. EAS technology refers to the tags that are placed on goods in stores and that trigger an alarm if they are removed from the store without deactivating the magnet that produces a field on the tag. In the present case, the bias magnet produces a strain-dependent magnetic field and so can provide information about the strain state of the substrate to which it is attached. These properties may eventually be useful in the monitoring of marine risers. It is important to note that the geometry and dimensions of the strain sensor presented here are particularly well suited to use in marine risers. This had motivated the suggestion to use magnetoelastic strain sensors for monitoring risers in a Brazilian patent [21]. This paper presents experimental results for the application of these sensors on steel surfaces as well as a model for understanding the shapes of the curves observed.

**Figure 2:**
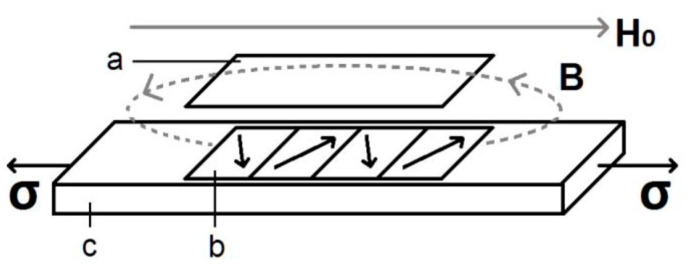
Schematic representation of a wireless strain sensor based on the Δ*E* effect: (**a**) resonator, (**b**) transducer, and (**c**) test object. Adapted from [22].

A schematic representation of our sensor is shown in Figure 2. A transducer (b) is glued to the test object (c). The bias field H_0_ determines the operating point on the Δ*E* curve of the resonator (a). In an operational device, the bias field might be supplied by a permanent magnet or by a Helmholtz coil. Application of a stress to the test object leads to a variation in the magnetic induction B, which modifies the resonant frequency of the resonator (a).

Our group has shown how magnetic field annealing [22] of the amorphous strips used both as transducers and resonators can increase the device response. Thus, in Figure 2, the moments depicted indicate that the amorphous strip used as a transducer had been subjected to a field anneal in the plane of the ribbon and perpendicular to the long ribbon axis. This is also true for the resonator. The amorphous material used initially [22] has a magnetostriction of about 11 ppm. In order to ascertain the importance of a material with suitable magnetoelastic properties, a polycrystalline Fe–Al–B alloy with piezomagnetic coefficient d = 1.5 nm/A [23] was tested as the transducer [24], although this material was not field-annealed nor stress-annealed in our work. A most interesting result was the highly linear relation between resonant frequency and strain for a nonmagnetic brass test object [24].

In this paper, we present results for a sensor composed of a transducer made from a polycrystalline Fe–Al–B alloy, while the resonator is a field-annealed amorphous alloy. Of the low-cost Fe-based alloys, two (Fe–Ga and Fe–Al) show reasonable values of piezomagnetic coefficients (for Ga and Al contents of ∼20 at %) at low values of the magnetic field. In contrast to [24], where the test object was a brass bar and the curve of resonant frequency vs. strain was found to be linear, the test object here was a bar of SAE 1010 steel. The variation of the resonant frequency with strain was no longer found to be linear because the additional field from the ferromagnetic substrate led to the saturation of the resonator. Nevertheless, that variation was very large and showed that this sensor could be used to measure strains in steel objects after an appropriate calibration procedure. 

## 2. Materials and Methods

Field-annealed Metglas 2826MB3 (with composition approximately Fe_45_Ni_45_Mo_7_B_3_ in wt %) was shown [22] to function well as a resonator in this strain sensor. The annealing (315 °C, 1 h, and 500 Oe) of the amorphous ribbon was performed in the plane of the ribbon, perpendicular to the long axis of the ribbon. For the transducer, a strip of Fe–Al–B was used. The Fe–Al–B has a magnetostriction much larger than the amorphous alloy (~11 ppm). Fe–Al–B alloys and especially the one applied in the present study were extensively studied in previously works [23,25,26]. The Fe–Al–B plate used as a transducer in our sensor has a composition of 20% aluminum in the matrix and a total of 1.6% of B (in at %). The material is polycrystalline and has two phases. A homogenization heat treatment was applied during 48 h at a temperature of 1200 °C after the melting process. The Al matrix consists of a mixture of α (A2 structure) and Fe_3_Al (D0_3_ structure) phases. In addition, small amounts of the tetragonal phase Fe_2_B are found in the grain boundaries. More details on the alloy fabrication can be found in [23]. The saturation magnetic polarization *J_s_* = *μ*_0_*M_s_*, in which *M_s_* is the saturation magnetization, was measured to be ~1.6 T and the total saturation magnetostriction 45 ppm. This material achieved an improvement of the total saturation magnetostriction (*λ_total_* = 3/2 *λ_s_*) from 45 to 70 ppm after stress annealing. The respective improvement in the piezomagnetic coefficient was from 1.5 to 3.0 nm/A. However, the material used in the sensor fabrication was not stress annealed, because at that time we did not have access to this improved material. Other studies of stress annealed Fe–Al–B alloys with different Al compositions were also made in [26]. Strips of size 36 × 8 × 0.7 mm^3^ were cut from a small Fe–Al–B plate. 

**Figure 3:**
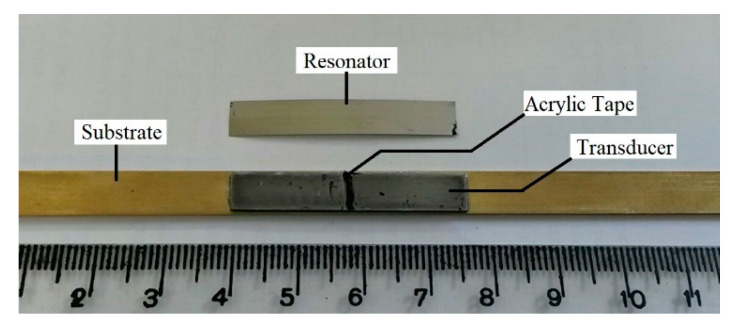
Sensor on the brass substrate [1,22]. The transducer was glued to the substrate while the resonator is held by tape located at the center of the strip where the amplitude of the longitudinal vibration is zero.

The structure of the sensor mounted on the brass substrate [24] is shown in Figure 3. The length L of the ribbon was arbitrarily chosen to be 36 mm, giving it a resonance frequency of about 61 kHz, and the width was chosen to be ≈ L/5, a relation that results in a single mode being excited [15]. The resonance frequency is f = (*E*/*ρ*(1 − υ))^1/2^/2L, where *E* is the Young modulus, ρ the density, and υ the Poisson ratio [27]. For the lowest frequency vibration, given previously, the resonator vibration is longitudinal with a node at the center of the strip. The frequencies and vibration amplitudes of the amorphous strips have been discussed extensively in [28]. The thickness of the Fe–Al–B strip was 0.7 mm, while the thickness of the amorphous ribbon used for the resonator was 30 µm. The same dimensions were used for the sensors employed with the steel strips. No attempt was made to optimize the sensor performance by varying sensor dimensions.

**Figure 4:**
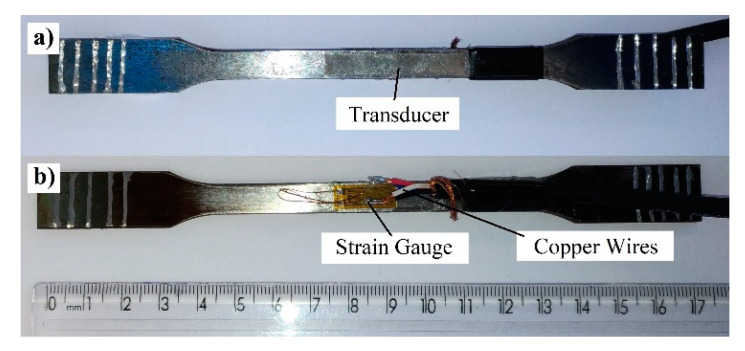
Sensor mounting on steel substrate [1]. In (**a**) the transducer is seen glued to the steel substrate, while in (**b**) the reverse side of the substrate is shown with a conventional strain gauge attached.

The mounting of the sensor is shown in Figure 3 and Figure 4. In Figure 3, the sensor is mounted on a brass substrate with the resonator positioned above the transducer and held in place by a narrow piece of acrylic tape located at the center of the transducer. For the lowest frequency vibration, the resonator vibration is longitudinal with a node at the center of the strip. The frequencies and vibration amplitudes of the amorphous strips have been discussed extensively [28]. Thus, the tape is affixed to the strip at the point where the vibration amplitude is null. In Figure 4, the transducer and strain gauge are seen to be mounted on opposite sides of the steel substrate. The magnetoelastic sensor was set up as in Figure 3 with the acrylic tape securing the resonator to the transducer. In initial measurements, the deformation was calculated from the advance of the EMIC (Instron) universal tester. These deformations were verified with the strain gauge.

Stress was applied to the strips of SAE 1010 steel with an EMIC universal tester, and deformations were calculated from the advance of this tester. Hysteresis curves were determined with a Globalmag hysteresis loop tracer. Sensor resonant frequencies were determined using an Agilent E5061B impedance analyzer. The impedance analyzer provided both the bias field and the excitation field for the experiment. 

**Figure 5:**
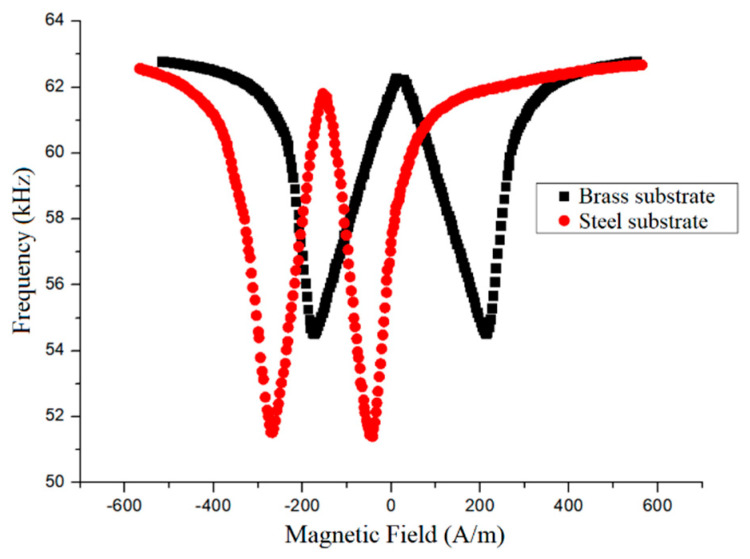
Compound ∆*E* curve for brass substrate (black) and thermally demagnetized steel (red) [1].

The first step in characterizing the sensors was to obtain the Δ*E* curve by measuring the resonant frequency (f) as a function of bias field. The presence of the transducer, a material that is magnetized by the external bias field, influences the measurement, so that the ∆*E* curve was obtained with both transducer and resonator in place and was referred to as a compound ∆*E* curve. These would be the only magnetic materials present in the case of the brass substrate. For the steel substrate, we must also consider the effect of the magnetization of the steel. The presence of the ferromagnetic substrate also influences the ∆*E* curve, as can be seen in Figure 5 where a residual magnetization has shifted the curve along the field axis. For both curves, the magnetic field was swept from −570 A/m to 570 A/m. For the curve associated with the brass substrate, the center of the ∆*E* curve was located at 11 A/m, indicating a small residual magnetization probably associated with the Fe–Al–B. In the case of the steel substrate, the center of the ∆*E* curve was located at −154 A/m. The shift in the curve is associated with a field produced by the residual magnetization of the substrate. 

It is important to note also that the vibration amplitude is largest at the minimum of the ∆*E* curve and decreases to either side. The magnetic field for which it is no longer possible to observe the resonance determines the range over which the sensor might be used. 

The bias field was chosen to be a magnetic field above the minimum in Figure 5, since the curve presents a greater slope in this region and therefore confers a greater sensitivity to the sensor. Experiments were performed in two ways. Initially, the Fe–Al–B transducer was glued to the steel rod with a cyanoacrylate adhesive, and the sample deformation was determined from the advance of the testing machine. To check those results, we also used an arrangement wherein the sample deformation was monitored simultaneously by both the magnetoelastic sensor and a strain gauge, both glued directly to the steel test piece with Henkel Locktite 496. The strain gauge had a resistance of 120 Ω and was connected to an HBM Quantum X data acquisition board. Data analysis was performed with the HBM Catman software. 

## 3. Model

We present here a model that can be used to describe the curves of resonant frequency as a function of deformation in these sensors. The Δ*E* curve describes the magnetic contribution to the resonant frequency and can be obtained from the piezomagnetic equations. The piezomagnetic equations, which consider the effect of the stress σ and the magnetic field H on the strain ε and the flux density B, can be simplified for a one-dimensional system, giving

(1) $$\varepsilon =\frac{\sigma }{E_{H}}+d_{33}H$$

(2) $$B=d_{33}^{*}\sigma +\mu^{\sigma }H$$

In these equations, *E_H_* is the Young modulus at constant magnetic field H and *μ^σ^* is the permeability at constant stress. The piezomagnetic coefficient *d*_33_ gives the variation in strain with magnetic field at constant stress, while $d_{33}^{*}$ represents the change in magnetic induction with stress at constant field. The two coefficients are equal for reversible exchanges of energy between the magnetic and mechanical systems. Eliminating the magnetic field from the strain equation, one obtains

(3) $${\frac{\partial \varepsilon }{\partial \sigma }|}_{B}=\frac{1}{E_{B}}=\frac{1}{E_{H}}\left(1-\frac{d_{33}^{*}d_{33}E_{H}}{\mu^{\sigma }}\right)$$

Because the resonant frequency f of the resonator is proportional to the square root of the Young modulus, one can show that

(4) $$\frac{f\left(E_{H}\right)}{f\left(E_{M}\right)}=\sqrt{\frac{E_{H}}{E_{B}}}={\left(\frac{1}{1+\frac{d_{33\;}^{*}d_{33}E_{B}}{\mu^{\sigma }}}\right)}^{1/2}={\left(1+\frac{d_{33\;}^{*}d_{33}E_{B}}{\mu^{\sigma }}\right)}^{-1/2}$$

The Young modulus *E_M_* corresponds to the case of a fixed domain configuration, the nonmagnetic case, and *E_B_* ≈ *E_M_*. The reduced magnetization is *m = M/Ms*. We use *B* = *μ_o_H* + *J*.

Using the relation between deformation and magnetic field for an isotropic material: $\varepsilon =\lambda_{s}\left(3cos^{2\;}\theta -1\right)/2$, where *θ* is the angle between *M* and the applied field and *M* = *M_s_*.cos θ, one obtains

(5) $${\left(\frac{d\varepsilon }{dH}\right)}_{\sigma }{}^{2}=d_{33\;}^{*}d_{33}=9\;\lambda_{s}^{2}\;\frac{M^{2}}{M_{s}^{4}}{\left(\frac{dM}{dH}\right)}^{2}=9\;\lambda_{s}^{2}m^{2}{\left(\frac{dm}{dH}\right)}^{2}$$

It is worth noting that for a reversible magnetoelastic behavior d_33_^*^ = d_33_ [29]. Finally one obtains for high permeability materials

(6) $$\frac{f\left(E_{H}\right)}{f\left(E_{B}\right)}={\left(1+\frac{9\;\lambda_{s}^{2}m^{2}\frac{dm}{dH}E_{B}}{J_{s}}\right)}^{-1/2}$$

In order to simulate the behavior of $\frac{f\left(E_{H}\right)}{f\left(E_{B}\right)}$ as a function of H, it is necessary to have an expression for *M(H)*. Our resonators were annealed in a magnetic field perpendicular to the long axis of the ribbon, so that the moments are ideally located in the plane of the ribbon, perpendicular to the long axis. *M(H*) for this ideal case is well known: *M* increases linearly up to saturation at the anisotropy field *H_A_*. However, the experimental Δ*E* curves are reproduced better if we assume rounding of *M(H)* near *H_A_*. Therefore, we represented *M* using a Langevin function, which is linear for low fields and rounded near *H_A_*:

(7) $$m=\frac{M}{M_{s}}=\;\left\{coth\;\frac{H}{H_{m}}-\frac{H_{m}}{H}\;\right\}$$

where *H_m_* is a parameter that depends on temperature with dimensions of magnetic field. Additionally,

(8) $$\frac{d\left(\frac{M}{M_{s}}\right)}{dH}=\frac{dm}{dH}=\frac{1}{H_{m}}\left\{-\frac{1}{\left(senh(H/H_{m}\right){)}^{2}}+\frac{H_{m}^{2}}{H^{2}}\right\}$$

Using these equations, one can calculate $\frac{f\left(E_{H}\right)}{f\left(E_{B}\right)}$ for different values of *H_m_* and verify that the total frequency variation in the Δ*E* curve depends upon *H_m_*, becoming smaller as *H_m_* increases. This behavior was used to obtain a value for *H_m_*. These calculations were done using the physical constants of the Metglas^®^ ribbon: *λ_s_* = 11 ppm, *J_s_* = 0.88 T, and *E_B_* = 100 GPa. The value of *H_m_* is a characteristic of the Metglas ribbon *H_m_* = 12.6 A/m.

The magnetic field value associated with the minimum in the Δ*E* curve can also be used to estimate the demagnetizing factor N for the resonator. The demagnetization factor fit was made such that the *H* value of the *f*(*E_H_*)/*f(E_B_*) curve minimum was coincident with the experimental result. The demagnetization factor obtained is N = 1.75 × 10^−4^. The maximum value of the susceptibility, the initial susceptibility for *H_m_* = 12.6 A/m, is χ_i_ = 1.86 × 10^4^. Using the resonator dimensions (l = 36 mm, w = 7.2 mm, t = 30 μm) and the susceptibility value, one can use the equations of Chen et al. [30] to estimate a value for N. This estimated value agrees with the value obtained from the Δ*E* curve.

Now we would like to consider the effect of stress on the *f(E_H_*)/*f*(*E_B_*) curves. The first issue to be treated is the effect of gluing the transducer (Fe–Al–B) on the substrate. The substrate and Fe–Al–B deformations are given by Equations (9) and (10):

(9) $$\varepsilon_{sub}=\;\frac{\sigma }{E_{sub}}$$

(10) $$\varepsilon_{FeAlB}=\;\frac{\sigma_{FeAlB}}{E_{FeAlB}}$$

where *E_FeAlB_* and *E_sub_* are the transducer and substrate Young modulus, *σ* is the applied stress, and *σ_FeAlB_* is the stress felt by the Fe–Al–B transducer. It is given by ${}_{FeAlB}=\frac{\frac{E_{FeAlB}}{E_{sub}}}{1+2\frac{E_{FeAlB}}{E_{sub}}\;\frac{t_{FeAlB}}{t_{sub}}}\;\sigma$ [24].

When stress is applied to the transducer (and perhaps the steel plate), the magnetic field experienced by the resonator will change because the transducer (and steel plate) are ferromagnetic materials. Therefore, due to the magnetoelastic coupling, their magnetization changes with the stress *σ*, and this magnetization field will combine with the bias field, and the sensor response will be different.

The magnetoelastic energy density for an amorphous material, where θ is the angle between the magnetization and the stress axis, can be written as

(11) $$E_{me}=-\sigma \cdot \varepsilon =-\lambda_{s}\cdot \sigma \left(3\cdot cos^{2}\theta -1\right)/2$$

When the stress axis is parallel or antiparallel to the magnetization, cos^2^ θ = 1 and,

(12) $$E_{me}=-\sigma \lambda_{s}$$

The magnetoelastic energy given by (12) gives rise to a variation in the magnetization (*M_s_*) and the minimum magnetic energy corresponding to *E_me_* in terms of magnetization variation is:

(13) $$E_{mag}=-\mu_{0}M_{s}\cdot H=-J_{s}\cdot H$$

Equating Equations (12) and (13), we have an expression Equation (14) for the magnetic field produced by the magnetostrictive material, Fe–Al–B and SAE 1010, due to the application of stress, that is:

(14) $$H=\frac{\;\sigma \lambda_{s}}{J_{s}}$$

Then, the magnetic field applied to the resonator will have 2 contributions given by Equations (15) and (16):

(15) $$H_{FeAlB}^{\prime }=M_{bias}^{FeAlB}+\frac{\sigma_{FeAlB}\cdot \lambda_{s}^{FeAlB}}{J_{s}^{FeAlB}}$$

(16) $$H_{substrate}^{\prime }=M_{bias}^{sub}+\frac{\sigma \cdot \;\lambda_{s}^{sub}\;}{J_{s}^{sub}}$$

The first terms of Equations (15) and (16) are the constant magnetizations of the Fe–Al–B and substrate generated by the bias field. The second terms of both equations are the magnetic field contributions triggered by the application of stress (Equation (14)), a consequence of the magnetoelastic coupling of the magnetic materials.

Therefore, the field applied to the resonator can be written as:

(17) $$H^{\prime }=\;\alpha H_{FeAlB}^{\prime }+\beta H_{sub}^{\prime }$$

The parameters *α* and *β* determine the contribution of the fields $H_{FeAlB}^{\prime }$ and $H_{sub}^{\prime }$ to the transducer and substrate demagnetization fields, respectively. The values of *α* and *β* are ≤1 and positive. The material properties used in the simulation were *λ_FeAlB_* = 2/3 *λ_total_* = 30 ppm and *J_s_^FeAlB^* = 1.6 T, and *t_sub_* and *t_FeAlB_* are the thickness of the substrate and Fe–Al–B.

## 4. Results

**Figure 6:**
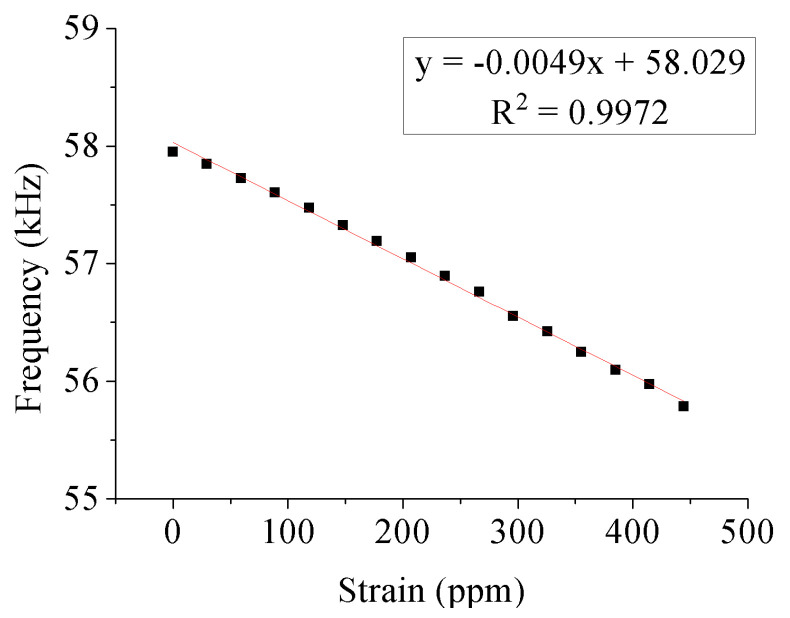
Magnetoelastic sensor resonant frequency vs. strain for a nonmagnetic brass substrate [24].

Previous measurements [24] on nonmagnetic brass substrates resulted in linear curves of resonant frequency vs. substrate deformation. This result is reproduced from Figure 3a of [24] in Figure 6. The value of R^2^ = 0.9972 shows that the curve is linear to a very good approximation.

**Figure 7:**
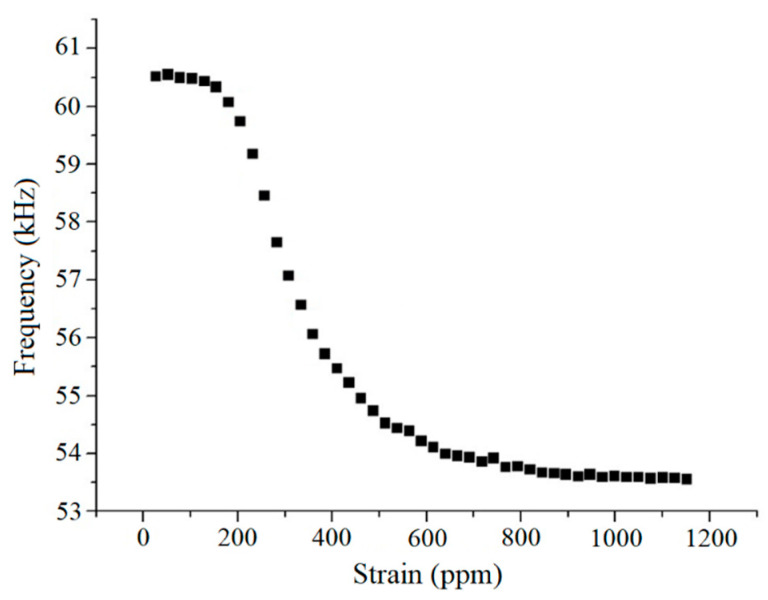
Sensor resonant frequency vs. sample strain for SAE 1010 steel substrate, as calculated from the advance of the testing machine. From [1].

Returning now to the experimental data, the compound ∆*E* curve for the steel substrate, shown in Figure 5, has large linear regions and might lead one to expect a linear relation between resonant frequency and sample deformation. However, there are several behaviors that can be observed, depending upon the bias field. The curve of resonant frequency vs. deformation is shown in Figure 7 for one of the steel substrates. The center of the compound ∆*E* curve in this case was located at 31 A/m. The remarkably large variation in the resonant frequency is about 7 kHz for a total sample strain of about 1100 ppm. In this case, the relation between resonant frequency and sample deformation is not linear as a whole, although there is a region presenting some linearity. The initial resonant frequency is practically constant from ~0 to 150 ppm. Still, the change observed in the linear region (200 to 400 ppm) is very pronounced. Moreover, the frequency variation is monotonic, always decreasing as the strain increases, but the frequency has essentially stopped changing by the time the strain reaches ~800 ppm.

The resonant frequency vs. advance time of the testing machine was also compared with the substrate deformation as measured by a strain gauge. The center of the compound ∆*E* curve was located at 292 A/m in this case. The response of the strain gauge was nearly linear, confirming that the steel substrate was deformed at a nearly constant rate in the tester. The resonant frequency presented a behavior similar to that seen previously. However, the total frequency change in this case was somewhat less than 2 kHz, confirming that the frequency shift may be sensitive to the choice of bias field or, at least, to the relation between the bias field and the field associated with the center of the compound ∆*E* curve. The bias field in this case was 507 A/m.

## 5. Discussion

We now wish to consider the two cases of a brass substrate and a steel substrate in terms of the model.

Case 1–Substrate: nonmagnetic brass 

For this case, the field acting on the transducer is H′ given by Equation (18).

(18) $$H^{\prime }=\alpha \left[M_{bias}^{FeAlB}+\frac{\frac{E_{FeAlB}}{E_{sub}}}{1+2\frac{E_{FeAlB}}{E_{sub}}\;\frac{t_{FeAlB}}{t_{sub}}}\frac{\lambda_{s}^{FeAlB}}{J_{s}^{FeAlB}}\;\;\sigma \right]$$

**Figure 8:**
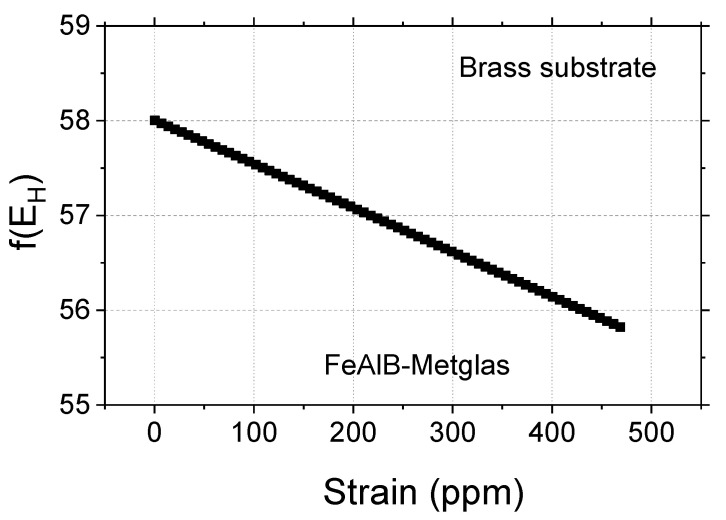
*f*(*E_H_*) as a function of strain. The transducer is the Fe–Al–B strip, the substrate is brass, and the resonator is a Metglas ribbon.

The strain of the testing machine is obtained dividing the applied stress by the Young’s modulus of the substrate. The Fe–Al–B and substrate thicknesses are equal. The additional materials properties are *E_FeAlB_* = 100 GPa and *E_sub_* = *E_brass_* = 97 GPa. The free parameters of the simulation are $M_{bias}^{FeAlB}$, *α*, and *m_bias_*. The experimental result was shown in Figure 6 above, and the value of the experimental applied bias field is close to 11 A/m. The simulation was carried out using *f(E_B_*) = 63 kHz and Equation (18). Using parameters with the values of $M_{bias}^{FeAlB}=260$ A/m, *α* = 0.008, and *m_bias_* = 0.18 (*J_sensor_* = 0.158 T), the experimental result can be reproduced. This result is shown in Figure 8 for the same range of deformations as the results of Figure 6.

The small *α* value is expected since the Fe–Al–B strip is short and wide and due to demagnetization factors reduces both fields associated to the Fe–Al–B transductor: the magnetization field due to the applied field and the other caused by the magnetoelastic coupling. The relationship of *α* with the demagnetization factor N_x_ is that *α* decreases if N_x_ increases (high demagnetization effect). For the fixed values of m = 0.18, if $M_{bias}^{FeAlB}$ and *α* are varied around these values the *f*(*E_H_*) curve dislocates to lower values keeping the magnitude of Δf constant.

Case 2–Substrate: magnetic SAE 1010 steel

For case 2, the experimental curve of *f(E_H_*) vs. ε was shown in Figure 7 above. The center of *f*(*E_H_*) vs. H curve is at 31A/m. The field *H*′ was calculated using Equation (17) and we considered *f*(*E_B_*) = 60.5 kHz. This steel has about 1 at % of nonmagnetic impurities, almost pure iron, so that *J_s_^SAE^* ≈ 2.0 T is a reasonable approximation. The value employed of *λ_SAE_* = 1.7 ppm is reasonable, as the magnetostriction of pure iron is −7 ppm [31] and tends to increase to positive values as nonmagnetic elements are added. For example, Fe–3Si (3 at %) has λ_s_ ~7 ppm [31], and therefore the SAE 1010 should have a magnetostriction in between that of pure iron and Fe–Si.

**Figure 9:**
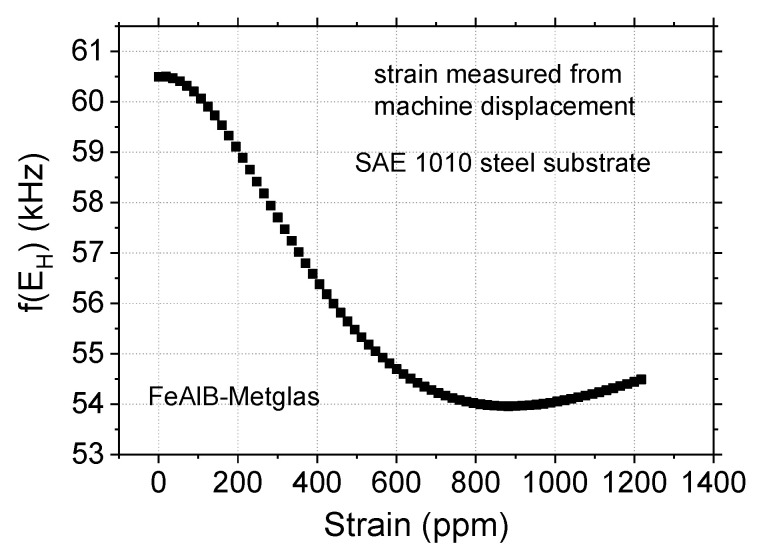
Resonant frequency vs. deformation for sensor with steel substrate.

The curve *f*(*E_H_*) vs. ε resulting from the simulation of the experimental curve in Figure 7 is shown in Figure 9 up to strains of 1200 ppm. The simulation reproduces the experimental curve well up to a strain of about 800 ppm. This curve is not linear as were those obtained on the brass substrates because the additional field created by the steel substrate saturates the resonator.

The fitting provided the values of $M_{bias}^{FeAlB}=$400 A/m, *α* = 0.009, $M_{bias}^{sub}=$20 A/m, *β* = 0.13 and *m_bias_* =−0.17 (*J_sensor_* = −0.15 T). The value of *m_bias_* is negative. This may mean that the sensor could have a negative remanence overlapping the magnetization generated by the bias field. The value of *α* is almost the same as the one obtained for the brass substrate and is coherent since the size of the Fe–Al–B strip did not change. The Fe–Al–B magnetization value is higher than the value obtained in the case of the brass substrate. That is consistent since the Fe–Al–B strip senses the bias and substrate fields and the first is higher than in the brass case. $M_{bias}^{sub}\ll \;M_{bias}^{FeAlB}$, which could indicate that the initial susceptibility of SAE steel is lower than that of Fe–Al–B. In addition, *β* > *α* is expected since the steel substrate is longer than the Fe–Al–B strip and they have the same width. The variations in *f*(*E_B_*) from one experiment to another are attributed to small differences in the Metglas ribbon length, which affects the demagnetization factor.

## 6. Conclusions

This work has shown that the wireless magnetoelastic sensor functions with a steel substrate, although the relation between the resonant frequency of the resonator and the strain is not linear, but rather sigmoidal in shape, or even more complex. The good signal-to-noise ratio observed was somewhat surprising since the sensor mass is so much smaller than the substrate mass and the substrate is ferromagnetic. Nevertheless, the high permeability of the sensor material easily compensates the elevated mass of the substrate. A model calculation has reproduced the general form of the frequency vs. deformation curves. Resonant frequency variations of up to 7 kHz have been observed, indicating that the sensitivity of the sensor is excellent even when used on a ferromagnetic substrate. However, the exact shape of the compound ∆*E* curve, the value of the bias fields, and the possibility of magnetic saturation have a lot to do with the resonant frequency curve, which is eventually obtained. The sensor sensitivity could be increased by using a stress annealed Fe–Al–B alloy, because this process would cause an improvement of the respective material piezomagnetic coefficients. The decrease of the transducer and sensor demagnetizing factors could also have a positive effect on the sensor sensitivity.

It is also important to point out that the sensor described here has a geometry that is very favorable for measuring the stress on the steel wires of the tensile armor of a marine riser. This fact was pointed out in a Brazilian patent [21]. If one were to place a sensor on each wire of the tensile armors, then it would be possible to follow in detail the stress on the riser. This would only be possible for the thin sensors as we have described them. It has been estimated [32] that the maximum thickness of a sensor placed between layers of tensile armor would be about 0.7 mm. The transducer used here has a thickness of 0.7 mm, but this value could be reduced by hot rolling, so that the whole sensor package could be accommodated in the space of 0.7 mm.
